# The roles of family physicians during a pandemic

**DOI:** 10.1177/08404704221112311

**Published:** 2023-01

**Authors:** Maria Mathews, Leslie Meredith, Dana Ryan, Lindsay Hedden, Julia Lukewich, Emily G. Marshall, Richard Buote, Lauren Moritz, Sarah Spencer, Shabnam Asghari, Judith B. Brown, Thomas R. Freeman, Paul S. Gill, Rita K. McCracken, Madeleine McKay, Bridget Ryan, Shannon L. Sibbald, Stephen Wetmore, Eric Wong

**Affiliations:** 1 6221 Western University, London, Ontario, Canada.; 2 1763 Simon Fraser University, Burnaby, British Columbia, Canada.; 3 7512 Memorial University, St. John’s, Newfoundland and Labrador, Canada.; 4 3688 Dalhousie University, Halifax, Nova Scotia, Canada.; 5 7512 Memorial University, St. John’s, Newfoundland and Labrador, Canada.; 6 12366 University of Toronto, Toronto, Ontario, Canada.; 7 12358 University of British Columbia, Vancouver, British Columbia, Canada.; 8 103987 Doctors Nova Scotia, Dartmouth, Nova Scotia, Canada.; 9 6221 Western University, London, Ontario, Canada.; 10 Thames Valley Family Health Team, London, Ontario, Canada.; 11 St. Joseph’s Health Care London, London, Ontario, Canada.

## Abstract

Family physicians play important roles throughout all stages of a pandemic response; however, actionable descriptions outlining these roles are absent from current pandemic plans. Using a multiple case study design, we conducted a document analysis and interviewed 68 family physicians in four Canadian regions. We identified roles performed by family physicians in five distinct stages of pandemic response: pre-pandemic, phased closure and re-opening, acute care crisis, vaccination, and pandemic recovery. In addition to adopting public health guidance to ensure continued access to primary care services, family physicians were often expected to operationalize public health roles (eg, staffing assessment centres), modulate access to secondary/tertiary services, help provide surge capacity in acute care facilities, and enhance supports and outreach to vulnerable populations. Future pandemic plans should include family physicians in planning, explicitly incorporate family physician roles, and ensure needed resources are available to allow for an effective primary care response.

## Introduction

Family physicians and other primary care providers play important roles in pandemic response and recovery. They must adapt to new roles and protocols as evidence about the spread, detection, symptoms, sequelae, and treatment of the disease evolves over time.^1^ Family physicians may be called upon to provide surge capacity in emergency rooms, hospitals, and long-term care homes that may be overwhelmed by cases^1-4^ while continuing to provide essential primary care services.^5^

Public health reports, including those following SARS and H1N1, outline the goals for primary care pandemic response plans (eg, contribute to screening, testing, treating, surge capacity, and vaccination),^1,3,6^ but do not explicitly include actionable plans describing family physician roles. The limited number of primary care-oriented pandemic response checklists available prior to COVID-19 were clinic-focused and without mechanisms to consolidate individual family physician roles across work settings, coordinate across different clinics, or integrate with other health sectors or regional organizational structures.^8-10^

The purpose of this study was to identify the roles of family physicians during different stages of the COVID-19 pandemic. Roles refer to the specific tasks and/or responsibilities that family physicians are asked or required to fulfill. These roles are expected to be temporary and stop once the pandemic is over and “normal” operations resume. Identifying the various roles performed by family physicians is an integral step to ensuring that required resources and training are incorporated into future pandemic preparedness.

## Methods

As outlined in a published protocol,^11^ we conducted a multiple case study of four regions in Canada: the Vancouver Coastal Health region in British Columbia (BC), the Eastern Health region in Newfoundland and Labrador, the province of Nova Scotia, and Ontario Health West. The four regions each include urban and rural communities and cover the continuum of health services.^2,12-14^ Each region has had different rates of COVID-19 cases, hospitalizations and deaths, and used primary care providers in different ways during the pandemic. Primary care organizations and policies also vary by region.

In each case, we relied on two sources of data: a document analysis and semi-structured qualitative interviews with family physicians. From March 2020 to April 2022, we identified publicly available policy documents impacting family physician roles in the pandemic. From October 2020 to June 2021, we conducted semi-structured qualitative interviews^15^ by phone or Zoom (Zoom Video Communications Inc.) with family physicians in each region. To be included in the study, family physicians must have had an active licence to practice in 2020 in their region. We excluded post-graduate medical residents, physicians on temporary pandemic-related practice licences, and physicians in solely academic, research, or administrative roles.

During the interview, we asked participants to describe the various pandemic-related activities they performed over different stages of the pandemic and the facilitators and barriers they experienced in performing these roles. We also asked about other potential roles that family physicians could have filled.

We used content analysis^16,17^ to identify the roles performed by family physicians and the corresponding stage or period during the pandemic in which they were performed in both the collected documents and the transcripts. We presented an initial list of pandemic stages and pandemic roles to our broader study team as well as to family physicians, public health officials, healthcare administrators, and policy makers to confirm the description of these stages and roles.

We obtained approval from the research ethics boards at Simon Fraser University and the University of BC, the Health Research Ethics Board of Newfoundland and Labrador, Nova Scotia Health, and Western University. Participants provided informed consent verbally before interviews were scheduled.

## Results

We interviewed 68 family physicians (Table 1). We identified five distinct pandemic stages and the family physician role in each stage: (1) pre-pandemic, (2) phased closure and re-opening, (3) acute care crisis, (4) vaccination, and (5) pandemic recovery (Table 2). There was not always a clear demarcation between these stages, and they did not always occur in a linear sequence. Stages also differed in regards to start dates and duration between regions. The phased closure and re-opening, acute care crisis, and vaccination stages overlap, with family physicians carrying out the roles associated with all three stages at the same time. The following section will define the pandemic stage and describe activities that family physicians perform or are expected to perform.

**Table 1.** Characteristics of study participants (n = 68).

	n (%)
Province	
British Columbia	15 (22.1)
Ontario	20 (29.4)
Nova Scotia	21 (30.9)
Newfoundland and Labrador	12 (17.6)
Gender^a^	
Men	27 (39.7)
Women	41 (60.3)
Practice type	
Fee-for-service	22 (32.4)
Alternative payment plan^b^	46 (67.6)
Hospital privileges	
No	18 (26.5)
Yes	49 (73.5)
Community size^c^	
Rural	20 (29.4)
Small urban	1 (1.5)
Urban	44 (64.7)
Mix	3 (4.4)
Years in practice (mean [standard deviation])	16.9 (9.72)

^a^Gender was asked as an open-ended question.

^b^Alternate payment includes all non-fee-for-service or enhanced fee-for-service payment types.

^c^Rural ≤ 10,000 population, small urban = 10,000 - 99,999 population, and urban ≥ 1,000,000.

**Table 2.** Family physicians pandemic roles and activities by pandemic stage in four regions in Canada.

Pandemic stages					
Pre-pandemic	Phased closure and re-opening	Acute care crisis	Vaccination	Pandemic recovery	Family physicians’ pandemic roles and activities
✓	✓	✓	✓	✓	Provide leadership at clinic, institutional, or regional levels
✓	✓				Implement infection prevention and control
✓	✓				Screen patients (active and passive screen)
✓	✓				Test patients or refer patients for testing
✓	✓				Advise patients on isolation/quarantine
✓	✓				Monitor COVID-19 patients recovering at home
	✓				Use special procedures to access supplies (swabs and personal protective equipment)
	✓				Educate patients about COVID-19, and infection control
	✓				Triage patients requiring in-person care
	✓			✓	Modulate referrals to elective, specialist, surgical care; imaging, and/or lab testing
	✓				Use virtual and telephone visits
	✓				Direct patients to assessment centres/telephone triage lines
	✓				Staff assessment centres
	✓				Identify and support vulnerable patients (eg, frailty and addictions)
	✓				Support travellers requiring screening/testing/isolation
	✓				Support community-based residential facilities experiencing outbreaks
	✓				Update care plans/goals for long-term care residents
		✓			Staff field hospitals or mobile health clinics
		✓			Assist with capacity in emergency departments and hospitals
			✓		Identify and support patients prioritized for vaccination
			✓		Address vaccine hesitancy
			✓		Staff vaccination centres and mobile clinics
				✓	Participate in evaluations of pandemic response

Phases may not occur in a linear sequence and not all phases will occur in each region or pandemic. The phased closure and re-opening, acute care crisis, and vaccination stages may overlap.

### Pre-pandemic stage

During the pre-pandemic stage, an influenza-like illness was identified and public health officials began to track cases and warn family physicians and other healthcare providers to be aware of potential cases. Given the novelty of the virus, there was little specific information about the condition, and as a result, family physicians relied on broad protocols for detection, treatment, and infection prevention and control. Diagnostic tests were not widely available, so detection was based on an evolving cluster of symptoms. Treatment was poorly understood and vaccines were in development.^18^ During this stage, family physicians were expected to provide leadership in organizing pandemic response. In the COVID-19 pandemic, the pre-pandemic stage occurred from January to March 2020.

### Phased closure and re-opening stage

During the phased closure and re-opening stage, there were restrictions on societal activities that varied in severity depending on number of local cases, potential for widespread infection, and ability of the healthcare system to meet demand for services.

The phased closure and re-opening stage of the pandemic began in mid-March 2020 and continued until April 2022, when many of the more restrictive public health directives were lifted. Given the likelihood of interacting with symptomatic individuals, family physicians used special procedures to access supplies. In response to closures and public health orders to limit in-person interactions, family physicians rapidly adopted new routines for providing care and operating their practices, such as using virtual or telephone visits. As a result, family physicians were required to quickly identify and adopt a system to triage and prioritize patients appropriate for in-person visits. The isolation and altered access to health and support services increased risks for vulnerable patients, requiring family physicians to provide additional supports for individuals with mental health and substance use issues, individuals experiencing homelessness, and frail elderly individuals.

During this stage, pandemic-specific facilities were established, such as dedicated telephone triaging lines, COVID-19 assessment centres, and clinics for respiratory illnesses. Family physicians were called upon to staff these facilities and/or direct patients to them (the latter requiring them to stay abreast of the latest screening protocols, therapies, and other resources in their region, and relay and explain this information to patients). During this stage, family physicians were also called upon to educate their patients as well as their broader communities about COVID-19 and explain and justify public health measures. Family physicians also monitored symptoms and provided healthcare advice to individuals who contracted COVID-19 (the majority of whom would recover in the community and not require acute care services) or who were isolating due to travel or coming into close contact with individuals who tested positive for the virus.

Limited in-person specialist appointments and increased pressure on hospitals also meant that family physicians had to modulate demand for secondary and tertiary care services to reduce the pressure on the hospital system and diagnostic services. As case numbers rose and the burden on hospitals increased, family physicians had to refer judiciously to limit demand for hospital-based services. Similarly, family physicians may have had to limit requisitions for routine lab testing for vulnerable patients if public health measures (social distancing and capacity limits) required patients to queue for long periods (often outside).

Residential facilities such as group homes or long-term care were particularly susceptible to outbreaks of COVID-19, especially during the early waves of the pandemic. Family physicians were called upon to assist these facilities when outbreaks occurred. Given the high susceptibility of residents in long-term care homes to COVID-19, family physicians were advised to update care plans and goals for their patients in these facilities.

### Acute care crisis stage

During the acute care crisis stage, hospitals were overwhelmed and unable to cope with the demand for services. In some locations, mobile health units or field hospitals were constructed to add capacity. Moreover, hospital staff may be exposed to the illness and need to isolate.

During the COVID-19 pandemic, the acute care crisis stage overlapped with the phased closure and re-opening and vaccination stages. Additionally, the COVID-19 acute care crisis stage was reached only in a few provinces, and during different waves and time periods in the pandemic. Regions in Ontario experienced an acute care crisis stage in January and April 2021 and in January 2022. Faced with increased demand for services and human resource shortages, hospitals turned to family physicians to assist with hospital and emergency department staffing and to staff field hospitals.

### Vaccination stage

The vaccination stage began once a vaccine was approved and available for use. The vaccination stage continued with each additional dose or booster. The vaccine stage overlapped with other stages in the pandemic. The vaccination stage began in December 2020 during the COVID-19 pandemic at which time the initial dose of the vaccine became available for high priority groups.

During the vaccination stage, family physicians helped identify and prioritize patients in their practice who were eligible to receive the vaccine. Family physicians needed to monitor the timing of other routine vaccinations that may impact patients’ eligibility for the vaccination of the pandemic-causing illness. In addition to educating their own patients about the vaccine and addressing vaccine hesitancy, family physicians may have been asked to contribute to public education campaigns in their communities. Family physicians were also be called upon to staff mass vaccination centres, help with vaccination in long-term care facilities, and staff mobile or pop-up clinics designed to provide access for hard-to-reach populations.

### Recovery stage

The pandemic recovery stage will begin once the widespread risk of the pandemic subsides. At the time of writing, Canada had not entered the pandemic recovery stage, although most pandemic measures had been loosened or discontinued in each region. During this stage, based on evidence from large-scale disasters such as Hurricane Katrina,^4,5^ family physicians should expect an increased demand for primary care services from patients who had delayed or forgone care during the pandemic (including among patients with pre-existing chronic conditions) as well as an increase in demand for mental health services. Given the backlog in services that were postponed during previous phases of the pandemic (eg, elective surgeries, screening, and diagnostic tests), family physicians may need to continue to modulate demand for hospital-based services, while resuming the provision of routine care. During this stage, there is a need to evaluate the pandemic response, which will contribute to the development of clearer pandemic plans for the future. It is imperative that family physicians participate in these evaluations to ensure primary care considerations are incorporated into future pandemic preparedness plans.

## Discussion

Through a comprehensive document analysis and interviews with family physicians, we identified five stages of pandemic response and the family physician roles associated with each stage. The World Health Organization (WHO) has identified four phases in pandemic response,^19^ which are incorporated into national and provincial/territorial influenza pandemic planning documents in Canada.^1^Table 3 highlights how the pandemic stages in this study correspond to the phases in the WHO pandemic planning document. The stages of the WHO document relate to the detection of new virus mutations, the epidemiology of the spread of an influenza disease, and the coordination of global surveillance and public health measures. The five stages identified in the study provide more granularity about family physician roles during the pandemic at a health system level and can facilitate jurisdiction-specific (eg, national, provincial, and/or regional) planning and coordination of services, as well as material and policy supports.

**Table 3.** Stages in a pandemic response in relation to the WHO pandemic response phases.

Stages in pandemic response	WHO pandemic response phases^18^
	Interpandemic period
Pre-pandemic	Alert phase
Phased closure and re-opening	Pandemic phase
Acute care crisis	
Vaccination	
Pandemic recovery phase	Transition phase
	Interpandemic period

Our findings draw attention to the importance of family physicians and primary care in a pandemic. Canada’s pandemic response relied heavily on family physicians, highlighting the dependence of public health and acute care on this sector of the health workforce. While family medicine usually includes many public health functions, during a pandemic, family physicians are expected to take on additional public health roles such as staffing assessment and vaccination centres, and communicating and adapting public health directives for their patients and the general population, in addition to continuing to provide routine primary care services to patients. Similarly, acute care facilities rely on family physicians to provide surge capacity by working in emergency departments and/or other units and to reduce demand for hospital-based services by delaying referrals for elective procedures and/or facilitating the discharge of patients who could be cared for in the community. Notably, current pandemic plans do not direct other healthcare sectors to boost primary care capacity,^1,6-8^ despite the additional roles that family physicians are required to adopt during a pandemic. Family physicians may also need additional supports to cover patient care responsibilities if they or their clinic staff contract the disease or are required to isolate. Also, primary care funding models may impact family physicians’ ability to take on pandemic roles. Post-pandemic evaluations should examine whether other healthcare providers (eg, registered nurses and specialists) could take on roles such as staffing assessment centres, given that these roles do not require specific family medicine expertise.

Identifying roles of family physicians is also an important step in ensuring that resources needed to fulfill these roles are readily available in a future pandemic. For example, the availability of personal protective equipment from the earliest stages of a pandemic is essential if family physicians are expected to continue to operate their practices and provide services. Also, during the COVID-19 pandemic, the implementation of fee codes was integral to the rapid adoption of telephone and virtual visits.^20^ Study findings also highlight education and training needs for family physicians. For example, family physicians may need additional training (eg, courses and continuing professional development certifications) to ensure that they have the specific skills needed to provide pandemic leadership^21^ or conduct virtual visits.^22^

### Limitations

We conducted interviews between October 2020 and June 2021, before Canada had entered the pandemic recovery stage. At the time of writing, the COVID-19 pandemic was ongoing and additional roles may be identified in the future as the pandemic response evolves. However, we continue to gather data through our document review and validate findings with practicing family physicians and public health experts to ensure that we do not miss roles. Despite using maximum variation sampling and a number of recruitment methods, our data may not fully capture the roles and experiences of all family physicians. Our research team is currently examining the pandemic-related roles of other primary care professionals (eg, community-based pharmacists and primary care nurses) in order to capture the full scope of roles that were adopted within the primary care sector. Our study focused exclusively on the primary care sector. Similar data from other healthcare sectors (eg, acute care and laboratory services) as well as other ministries are needed to support a coordinated, integrated pandemic response plan.

## Conclusion

The primary care sector plays an important role in pandemic response. We identified key roles that family physicians performed during the COVID-19 pandemic over five distinct pandemic stages: pre-pandemic, phased closure and re-opening, acute care crisis, vaccination, and pandemic recovery. In addition to adopting activities to ensure that primary care services continued to be available, family physicians were responsible for operationalizing public health priorities (such as surveillance and vaccination) and ensuring that acute care facilities can respond to increasing demand for acute care services. Family physicians were also entrusted with responding to the needs of patient populations who are particularly at risk. Pandemic preparedness plans should ensure that the resources needed to enable family physicians to fulfill these roles are available in the event of a future pandemic to allow for an effective and coordinated primary care response.
